# Cyclin-dependent kinase modulates budding yeast Rad5 stability during cell cycle

**DOI:** 10.1371/journal.pone.0204680

**Published:** 2018-09-26

**Authors:** Masafumi Hayashi, Kenji Keyamura, Takashi Hishida

**Affiliations:** Department of Life Science, Graduate School of Science, Gakushuin University, Tokyo, Japan; Università degli Studi di Milano-Bicocca, ITALY

## Abstract

The DNA damage tolerance (DDT) pathway facilitates the bypass of the fork-blocking lesions without removing them through either translesion DNA synthesis or error-free damage bypass mechanism. The *Saccharomyces cerevisiae* Rad5 is a multi-functional protein involved in the error-free branch of the DDT pathway, and its protein level periodically fluctuates through the cell cycle; however, the mechanistic basis and functional importance of the Rad5 level for the cell cycle regulation remain unclear. Here, we show that Rad5 is predominantly phosphorylated on serine 130 (S130) during S/G2 phase and that this modification depends on the cyclin-dependent kinase Cdc28/CDK1. We also show that the phosphorylated Rad5 species at S130 exhibit a relatively short half-life compared with non-phosphorylated Rad5 moiety, and that the Rad5 protein is partially stabilized in phosphorylation-defective *rad5 S130A* cells. Importantly, the elimination of this modification results in a defective cell-cycle dependent Rad5 oscillation pattern. Together, our results demonstrate that CDK1 modulates Rad5 stability by phosphorylation during the cell cycle, suggesting a crosstalk between the phosphorylation and degradation of Rad5.

## Introduction

Endogenous and exogenous DNA-damaging agents constantly challenge the integrity of the genome. Eukaryotic organisms have evolved several repair mechanisms that repair DNA damage [1]. However, when replication forks encounter fork-blocking lesions, the resumption of replication only after removal of the fork-blocking lesions would not be practical, as the completion of DNA replication would depend on the repair efficiency. To circumvent this dependency, the DNA damage tolerance (DDT) pathway ensures completion of DNA replication by bypassing unrepaired DNA lesions without removing them, thereby allowing cells to continue growing [2–4].

In budding yeasts, the DDT pathway consists of at least two parallel branches, translesion DNA synthesis (TLS) and the error-free damage bypass, both of which are controlled by covalent ubiquitin modification of proliferating cell nuclear antigen (PCNA) [5]. Monoubiquitination of PCNA is mediated by heterodimers comprising Rad6 (E2; ubiquitin-conjugating enzyme) and Rad18 (E3; ubiquitin ligase), which promote the TLS pathway [5, 6]. This pathway employs specialized DNA polymerases for translesion synthesis that individually, or in collaboration, allow replication to continue past replication-blocking DNA lesions. In this context, monoubiquitination of PCNA plays a critical role in TLS polymerase recruitment and/or rearrangement at the fork [7, 8]. Alternatively, polyubiquitination of PCNA through Lys63-linked chains requires another E2-E3 complex, Ubc13 (E2)-Mms2 (E2 variant) and Rad5 (E3), in addition to Rad6-Rad18 [5, 9, 10]. This modification of PCNA presumably promotes the error-free bypass of DNA lesions, in which fork blocking lesions are bypassed by recombination-associated template switching using the undamaged sister chromatid as a template [11–13]. Although this process and the mechanisms of its regulation by poly-ubiquitination of PCNA remain unclear, Rad5 appears to possess key functions for template switching besides the ubiquitination of PCNA. Indeed, Rad5 is a member of the SWI/SNF family of ATPases and possesses a DNA helicase activity, which can lead to the regression of replication fork-like structures *in vitro* [14]. 2D-gel analyses of replication intermediates show that Rad5 is involved in the formation of X-shaped DNA structures between sister chromatids at stalled replication forks [15, 16]. Recently, Choi K, *et al*. demonstrated that a mutation of the helicase motif VI (*rad5-QD*), which displays mild sensitivity to methyl methanesulfonate (MMS), is sufficient for the poly-ubiquitination of PCNA and the formation of recombination-mediated X-shaped DNA structures [17]. Moreover, Rad5 can promote translesion synthesis through a direct interaction with Rev1 [18]. Thus, Rad5 is a multi-functional protein that is involved in other functions independent of the PCNA poly-ubiquitination. Considering that their activities contribute to replication fork rescue and genome stability, understanding their functional relationship and temporal regulation is important for clarifying the mechanisms by which their functions are integrated.

Phosphorylation can influence numerous functions of a protein, including catalytic activity, localization, stability, and/or protein-protein interactions. Recent phospho-proteomics studies have identified several phosphorylation sites within *S*. *cerevisiae* Rad5 [19–21]; however, the regulation and functional importance of its phosphorylation remain unclear. In this study, we examined the phosphorylation sites on Rad5 using gel mobility shift assay and identified that serine 130 (S130) is the primary phosphorylation site responsible for the observed migratory shift. We also demonstrated that impaired phosphorylation in *rad5 S130A* cells results in a defective cell-cycle-dependent Rad5 oscillation pattern due to an increased stability of Rad5. These results demonstrated that cell-cycle dependent Rad5 phosphorylation at S130 facilitates the protein turnover, suggesting a direct link between phosphorylation and its degradation.

## Materials and methods

### Yeast strains, plasmids, and growth conditions

All yeast strains used in this study are listed in Table 1. Standard genetic procedures were used for strain construction and medium preparation [22]. Yeast cells were routinely grown in YPD medium containing 0.003% adenine sulfate (YPDA). Yeast strains carrying each plasmid were grown in synthetic complete medium lacking leucine (SC-LEU). The *RAD5-13Myc* strain was constructed by introducing a Myc epitope coding sequence (from pFA6a-13Myc) into the 3’ end of the *RAD5* locus in frame [23]. The PCR fragment containing the native *RAD5* promoter and the coding region of *RAD5-Myc* amplified using genomic DNA of the *RAD5-Myc* strain were cloned into pUC19, and new *Nde*I and *Bam*HI sites were generated at the 5’ and 3’ end of *RAD5*, respectively, yielding pMU001. All serine to alanine or aspartic acid substitutions were generated from pMU001 by site-directed PCR mutagenesis. For the construction of a single copy *CEN6/ARS* plasmid (pRS415) carrying wild-type *RAD5-Myc* or its mutants, the *Pst*I fragments containing the promoter and their coding regions from the pMU001 derivatives were cloned into pRS415. For expression of the truncation alleles, all truncation alleles amplified by PCR were cloned into the *Nde*I/*Bam*HI sites of pMU001. All constructs were confirmed by DNA sequencing.

**Table 1:** *S*. *cerevisiae* strains used in this study.

Strain	Genotype	Source
BY4741	*MAT*a *leu2*Δ*0 ura3*Δ*0 his3*Δ*1 met15*Δ*0*	ATCC^a^
MH001	BY4741 *rad5*Δ::*KanMX*	[24]
MH002	BY4741 *RAD5-13myc*::*KanMX*	This study
MH003	MH001 *bar1*Δ::*URA3*	This study
MH004	MH002 *bar1*Δ::*URA3*	This study
MH005	MH002 *cdc28as1*	This study
MH006	BY4741 *rad5*^*S130A*^*-13myc*::*KanMX*	This study
MH007	BY4741 *rad5*^*S129A S130A*^*-13myc*::*KanMX*	This study
MH008	MH006 *bar1*Δ::*URA3*	This study
MH009	MH007 *bar1*Δ::*URA3*	This study
MH010	MH001 *cdc28as1*	This study

^a^ American type culture collection

### Yeast cell extract and western blotting

Yeast cell extracts were prepared from yeast cultures using the trichloroacetic acid method, as described previously [25]. Protein samples were separated using SDS-polyacrylamide gels or Phos-tag SDS-polyacrylamide gels containing 25 μM Phos-tag (Wako) and 50 μM MnCl_2_. Proteins were transferred to PVDF membranes. Phos-tag SDS-PAGE gels were incubated with western blotting buffer containing 50 mM EDTA prior to the transfer of proteins. Rad5, Rad53 and tubulin were detected with anti-c-Myc (Roche), anti-Rad53 (Santa Cruz) and anti-α-tubulin (SIGMA) antibodies, respectively. The bands were revealed by chemiluminescence (ECL Select, GE Healthcare). The intensity of each protein band was quantified using Image J software.

### Immunoprecipitation and phosphatase assay

Cells were resuspended in lysis buffer (50 mM Tris-HCl/pH 7.5, 150 mM NaCl, 1 mM EDTA/pH8.0, 0.1% NP-40, 10% Glycerol) supplemented with protease inhibitor cocktail (Roche). Cell suspensions were disrupted using a homogenizer (Bertin technologies) and then centrifuged at 15,000 rpm at 4°C for 20 min. The protein extracts were collected and incubated with anti-c-Myc agarose beads (SIGMA) at 4°C for 2 h. The beads were washed four times with IP Buffer (20 mM Tris-HCl/pH7.5, 50 mM NaCl, 10% Glycerol, 0.5% NP-40). The precipitated samples were then resuspended in phosphatase buffer and incubated either with λ-protein phosphatase (NEB) or with λ-protein phosphatase and phosphatase inhibitor (Roche) for 30 min at 37°C. Rad5 protein was visualized by Phos-tag western blotting using anti-c-Myc antibody.

### Sensitivity to MMS and HU, and UV light

Cells were grown overnight at 30°C and ten-fold serial dilutions of the cultures were spotted onto YPDA or SC-LEU plates with or without MMS or HU. For UV sensitivity assays, the plates were irradiated with UV light (GE-10, Toshiba). The plates were incubated for 3 days at 30°C.

### Mutation frequency at the *CAN1* locus

Mutation frequencies were determined, as described previously [26]. Briefly, cells were grown to early logarithmic phase in SC-LEU medium and treated with 0.1% of MMS for 1 h. Subsequently, cells were washed with 5% sodium thiosulfate, diluted and plated onto plates at an appropriate dilution to determine the total cell number (SC-LEU plates) and the number of Can^R^ mutants (SC-LEU-ARG plates containing 60 μg/ml canavanine). Plates were incubated at 30°C for 3 days. The mutation frequency was determined by dividing the number of Can^R^ colonies per milliliter by the number of viable cells per milliliter, and the average was determined for at least three independent sets of experiments.

### Cell-cycle arrest and cycloheximide treatment

We used *MATa bar1* strain and its derivatives for cell synchronization. Cells were grown to early-log phase at 30°C in YPDA media and treated for 2 h with 100 ng/ml of α-factor (SIGMA). To release cells from α-factor arrest, cells were washed twice with water and resuspended in fresh YPDA media containing 100 μg/ml pronase (SIGMA). Samples were collected at various times for FACS analysis and protein extraction. For cycloheximide chase, cells were grown to mid-log phase at 30°C, and then treated with cycloheximide (400 μg/ml). Samples for whole cell extracts were taken at indicated time points. The level of proteins was detected by Phos-tag western blotting.

### Flow cytometry

Fluorescence-activated cell sorting (FACS) analysis and microscopy were performed as described previously [27]. Briefly, cells were fixed in 70% ethanol, washed and resuspended in 50 mM sodium citrate pH7.5. Samples were treated with RNase A (0.25 mg/ml) for 1 h at 50°C, and then with proteinase K (1 mg/ml) for 1 h at 50°C. Cells were stained with propidium iodide (16 μg/ml) for 1 h at 4°C. The DNA contents of cells were analyzed with a Becton Dickinson FACSCanto II flow cytometer.

## Results and discussion

### Phosphorylation of Rad5 during the cell cycle

**Fig 1:**
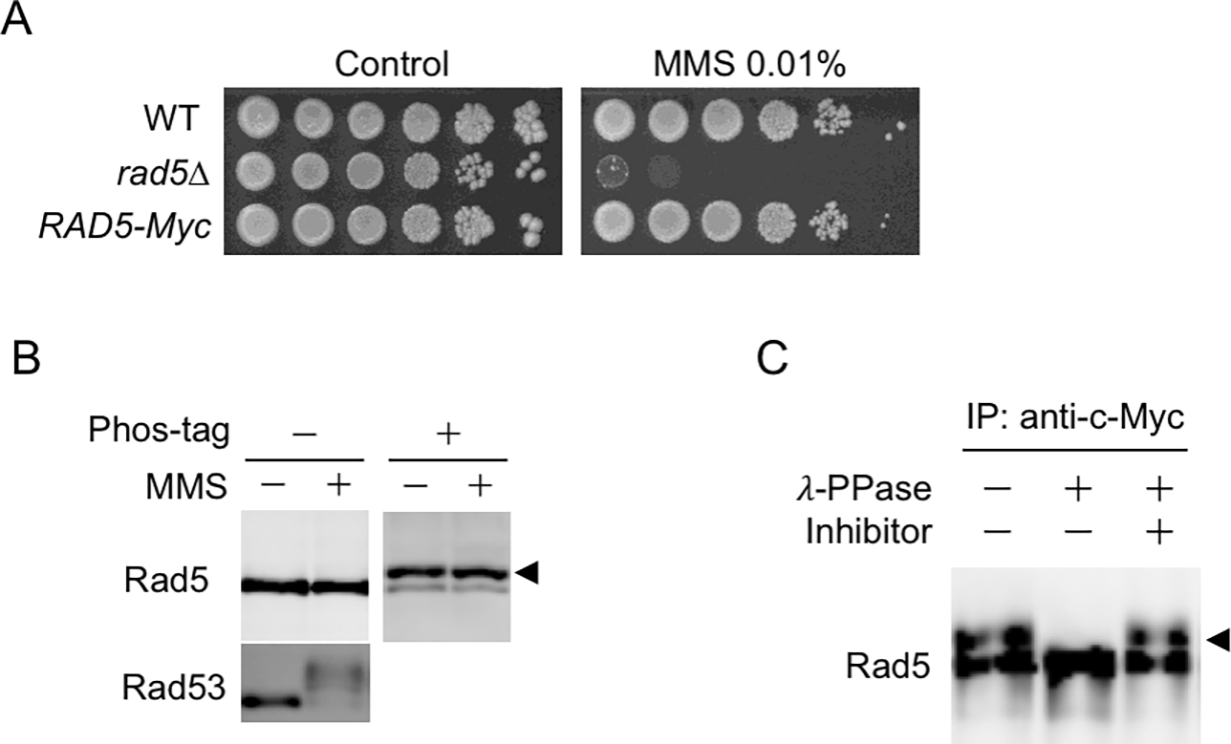
Rad5 phosphorylation under normal growth conditions. (A) MMS sensitivity of wild-type, *rad5Δ* and *RAD5-Myc* cells. Ten-fold serial dilutions of asynchronous cell cultures were spotted onto YPDA plates with or without MMS (0.01%) and incubated at 30°C for 3 days. (B) Detection of Rad5 phosphorylation by western blotting with anti-c-Myc antibody. Log-phase cultures of wild-type *RAD5-Myc* cells were either mock treated (-) or treated with 0.1% MMS (+) for 1 h. Protein extracts were then processed on a standard SDS-PAGE gel and on a Phos-tag SDS-PAGE gel. Rad5 and Rad53 proteins were detected with anti-c-Myc and anti-Rad53 antibodies, respectively. The slower migrating bands are indicated by arrow heads. (C) Immunoprecipitated Rad5 was treated with lambda phosphatase (or mock treatment) and phosphatase inhibitors prior to Phos-tag SDS-PAGE, followed by immunoblotting with anti c-Myc antibody.

In the present study, we investigated the possible Rad5 phosphorylation using a mobility shift assay in a yeast strain expressing the *RAD5-Myc* allele at its endogenous locus under control of the native *RAD5* promoter. C-terminal fusion to the 13×Myc epitope did not adversely affect Rad5 function because cells expressing Rad5-Myc showed the same sensitivity to MMS as wild-type cells (Fig 1A). Western blot analysis of asynchronous cell extracts showed that Rad5 migrated as a single band during electrophoresis irrespective of the presence of DNA damage (Fig 1B). However, analysis of Rad5 by Phos-tag SDS-PAGE and subsequent immunoblotting using anti-c-Myc antibody (Phos-tag western blotting), which slow down the migration of phosphorylated proteins, revealed that Rad5 migrated as two distinct bands and that the degree of the band shift did not change in the presence of MMS (Fig 1B). Lambda phosphatase treatment of immunoprecipitated Rad5 confirmed that protein phosphorylation was responsible for the formation of the slower-migrating species of Rad5 (Fig 1C). These results indicate that Rad5 is phosphorylated under normal growth conditions.

### Identification of Rad5 phosphorylation sites

**Fig 2:**
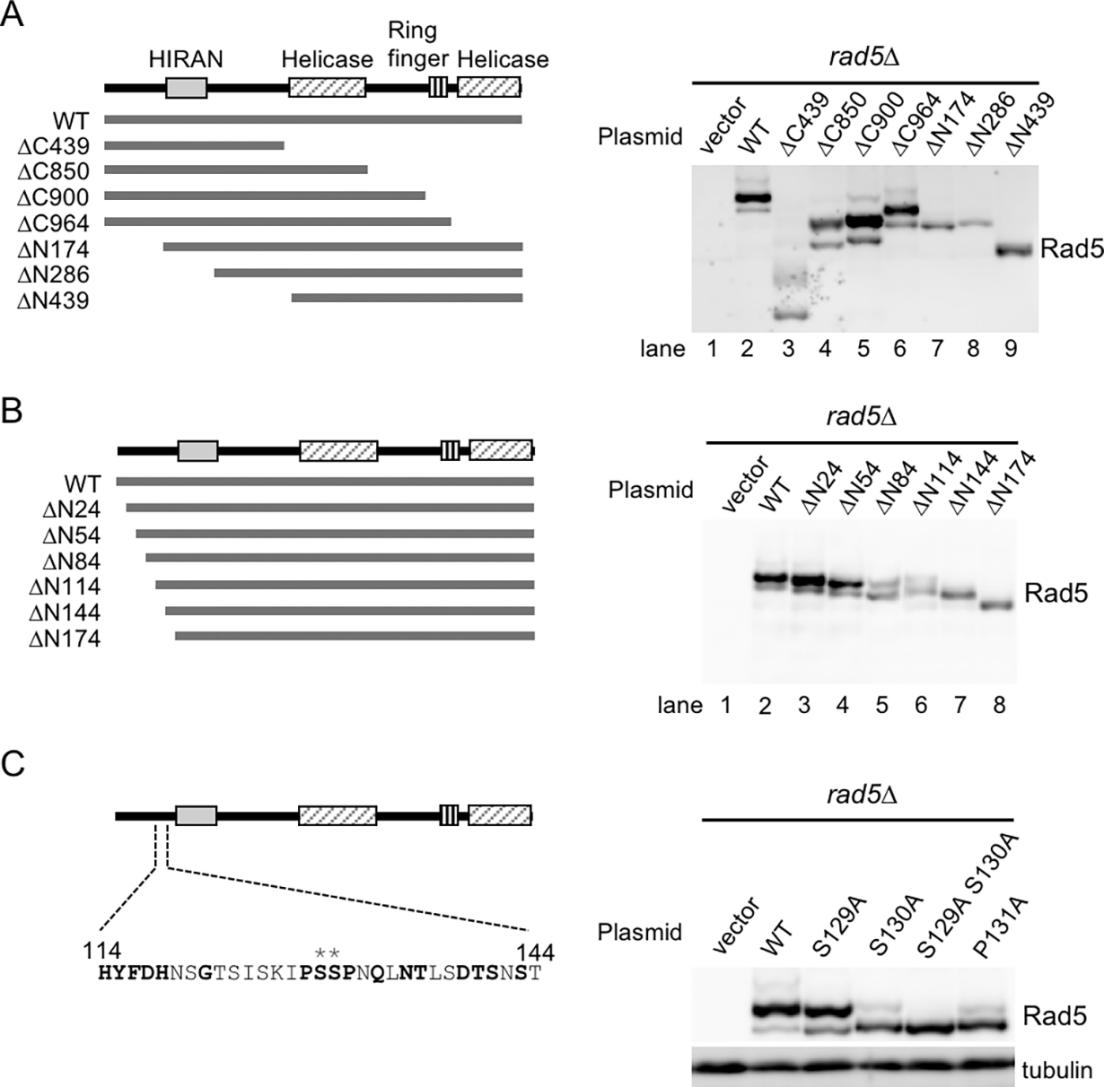
Identification of phosphorylation sites in Rad5. (A) Schematic representation of the Rad5 domains, including the HIRAN (176–285), helicase (440–848 and 1030–1164) and RING finger (913–961) domains, which are shown in the left panel. N- and C-terminal truncations of Rad5 shown in the left panel were constructed by fusion to a Myc epitope tag. *rad5Δ* cells were transformed with each of the *CEN/ARS* plasmids carrying wild-type or truncated alleles of *RAD5*. Expression of wild-type and truncated alleles from the endogenous *RAD5* promoter was confirmed by Phos-tag western blotting using c-Myc antibody (right panel). (B) N-terminal truncations ranging in size from 24 to 174 residues, shown in the left panel, were constructed by fusion to a Myc epitope tag. Expression of truncations was examined as described in (A) (right panel). (C) Phosphorylation state of Rad5-Myc with each single or double amino acid substitutions in the N-terminal region. A map of the Rad5 N-terminal region between amino acids 114 and 144 is shown in the left panel. Asterisks indicate the putative phosphorylation sites, S129 and S130. *rad5Δ* cells were transformed with plasmids carrying each *rad5* mutant. Conserved amino acid residues among budding yeast are shown in bold. Phosphorylation patterns were analyzed as described in (A) (right panel). Tubulin served as a loading control.

To determine the phosphorylation sites of Rad5, we generated truncations to the 5’ or 3’ end of the *RAD5* gene (Fig 2A) such that the truncations of the N- or C-terminus of *RAD5* were expressed under control of the native *RAD5* promoter as fusions to a Myc epitope on yeast *CEN/ARS* plasmids. Individual plasmids were introduced into the *rad5Δ* strain, and the band patterns of these truncation mutants were then analyzed by Phos-tag western blotting. The overall band patterns of wild-type Rad5-Myc expressed from the plasmid were similar to those of chromosomal *RAD5-Myc* cells. Among the various *rad5* truncation mutants, slower-migrating bands were detected for all C-terminal truncations (Fig 2A, lanes 3–6) but not for all N-terminal truncations (Fig 2A, lanes 7–9). Further truncation analysis of N-terminal regions indicated that a short region comprising amino acids 114–144 was responsible for a phosphorylation-dependent band shift (Fig 2B, lanes 7 and 8).

Previous mass spectrometry analyses of the yeast phosphoproteome identified several phosphorylated residues in Rad5 [19–21], and among these, two serine residues (S129 and S130) conserved among budding yeasts are localized in the N-terminal region spanning residues 114–144 (Fig 2C and S1 Fig). To further characterize the candidate phosphorylation sites, we generated two *rad5* mutants, one carrying an alanine substitution at S129 (*rad5-S129A*) and the other carrying an alanine substitution at S130 (*rad5-S130A*), and compared Rad5 phosphorylation in wild-type and mutant cells progressing in log-phase cultures. Rad5-S129A had a subtle change in the overall migration pattern of Rad5, as indicated by a slight increase in the amount of the faster migrating bands, whereas Rad5-S130A led to a considerable disappearance of the slower-migrating bands (Fig 2C). We also observed a faint band slightly above the main phosphorylation band in wild-type cells. This form of Rad5 represents doubly phosphorylated protein at both S129 and S130, because it is not detectable in *rad5-S129A* or *rad5-S130A* cells. Moreover, combining the two mutations eliminated all detectable slower-migrating bands of Rad5. These results suggest that Rad5 is phosphorylated predominantly at S130 and to a lesser extent at S129.

### *In vivo* functional analysis of phosphorylation-deficient or -mimic Rad5 mutants

**Fig 3:**
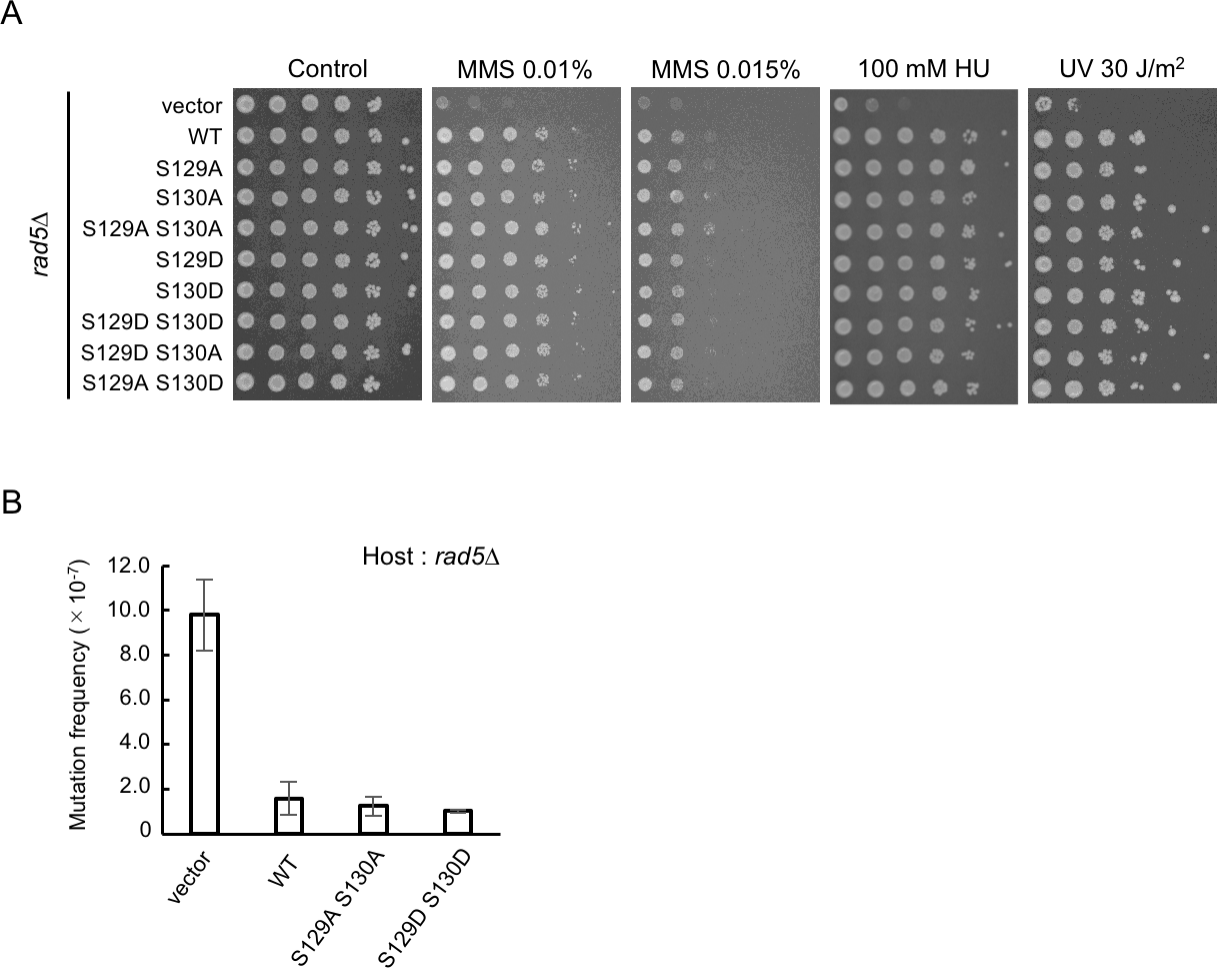
Functional analysis of Rad5 phosphorylation in DNA damage tolerance pathways. (A) DNA damage sensitivity of *rad5* mutants. *rad5Δ* cells carrying each of the indicated plasmids were 10-fold serially diluted and exposed to the indicated DNA-damaging agents, and plates were incubated at 30°C for 3 days. (B) Mutation frequencies at the *CAN1* locus in *rad5Δ* cells carrying the indicated plasmids. Cells were grown in SC-LEU medium at 30°C. Canavanine-resistant mutants were selected on synthetic complete medium lacking leucine and arginine, and containing canavanine. Error bars indicate the standard errors of the three independent experiments.

To examine whether Rad5 phosphorylation-deficient mutants affect error-free DDT activity *in vivo*, we transformed *rad5Δ* cells with plasmids harboring each of the phosphorylation-deficient mutations (*rad5-S129A*, *rad5-S130A*, and *rad5-S129A S130A*) and examined these mutants for sensitivity to methyl-methanesulfonate (MMS), hydroxyl urea (HU), or ultraviolet (UV) light exposure. Control experiments showed that *rad5Δ* cells harboring vector plasmid were extremely sensitive to MMS, HU, and UV light. In contrast, none of the phosphorylation-deficient mutants showed significant sensitivity to any of the treatments (Fig 3A). We next generated mutants in which S129 and/or S130 were replaced with an aspartic acid residue, which can, in some cases, mimic phosphorylation. These mutants also have wild-type sensitivity to DNA damaging agents (Fig 3A). To further investigate the possibility that Rad5 phosphorylation is important for genome stability, we measured mutation frequencies at the *CAN1* locus, at which any mutation that inactivates the arginine permease encoded by *CAN1* confers canavanine resistance (Can^R^). However, these phospho-deficient and–mimic mutants did not affect Can^R^ mutation frequencies (Fig 3B).

### Cell-cycle dependent phosphorylation of Rad5

**Fig 4:**
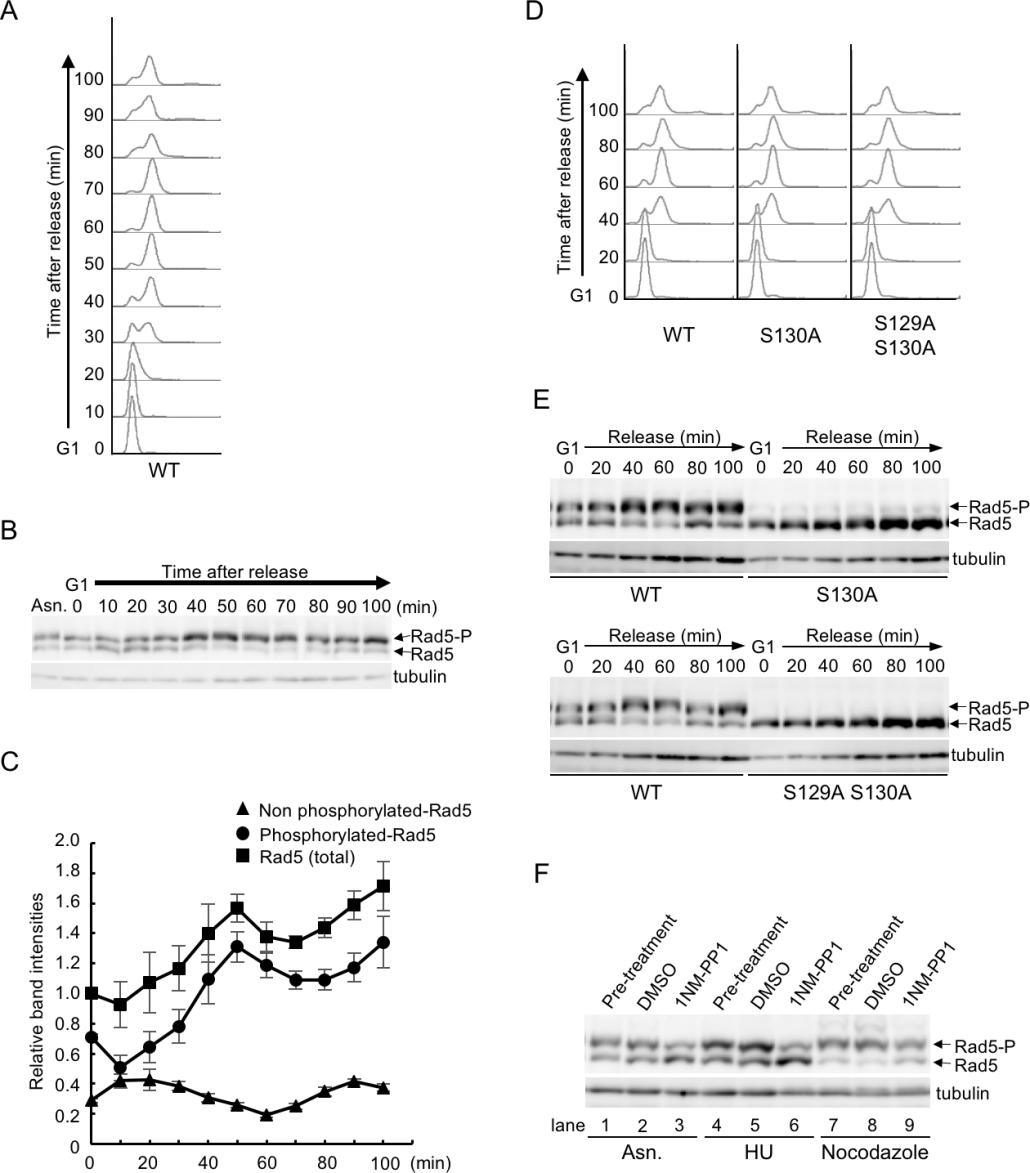
Elevated phosphorylation levels of Rad5 at S130 during the S to G2/M phases. (A, B) *RAD5-Myc* cells grown to early log phase at 30°C were arrested in G1 with α-factor for 2 h. Cells were then released into fresh YPDA medium. Samples from synchronized cell cultures were taken at the indicated time points after release from G1 block. Cell-cycle progression was determined by flow cytometry (A) and Rad5 protein was analyzed by Phos-tag western blotting (B). Tubulin served as a loading control. Asn denotes asynchronously growing cells. (C) Total amount of Rad5 is shown as relative band intensities together with the ratio of the relative amount of the two bands. Values are expressed relative to 1.00 for total amount of Rad5 at time zero. Error bars are derived from standard errors of the three independent experiments. (D, E) *RAD5-Myc*, *rad5-S130A* and *rad5-S129A S130A* cells were grown to log phase, arrested at G1 by α-factor, and then released into fresh YPDA medium. Cell cycle progression was analyzed by flow cytometry (D) and Rad5 protein levels were analyzed by Phos-tag western blotting (E). (F) *cdc28-as1 RAD5-Myc* cells were grown to log phase or arrested at S phase with 200 mM HU or at G2 phase with 20 μg/ml nocodazole. After 2 h, cultures (pre-treatment) were divided equally and treated with DMSO (mock) or 5 μM 1NM-PP1 for 1 h. Rad5 protein was analyzed by Phos-tag western blotting.

A previous study has shown that Rad5 expression oscillated over the cell cycle, with maximum levels in S phase [28]. To investigate whether Rad5 is phosphorylated in a cell-cycle-regulated manner, we arrested an early log-phase culture of yeast cells in G1 using α-factor and subsequently released the cells into a synchronous cell cycle. Samples were taken at each time point to evaluate Rad5 phosphorylation and to analyze the fluorescence-activated cell sorting (FACS) profiles. Consistent with the previous study, the abundance of Rad5 increased during the S/G2 phase (Fig 4A and 4B). In addition, we found that the phosphorylation bands with reduced electrophoretic mobility were detectable at G1 and immediately after release but accumulated when cells reached 2C DNA content (Fig 4A and 4B). To investigate whether the peak accumulation of phosphorylation bands merely coincides with that of the total expression levels of Rad5, we calculated the ratio between the relative densities of the slower-migrating (phosphorylated) and faster migrating (non-phosphorylated) bands of Rad5 for the cells released to the cell cycle. The level of phosphorylated Rad5 reached a maximum at 50 min after release from G1 arrest, which was slower than the non-phosphorylation peak window (10–20 min) (Fig 4C). These results suggest that the cell-cycle-dependent alternations in phosphorylated Rad5 levels represent a specific increase in Rad5 phosphorylation during S/G2 phase as well as an increase in its expression.

To address whether Rad5 phosphorylation affects cell-cycle-dependent protein oscillations, we examined the expression of Rad5-S130A and Rad5-S129A S130A throughout the cell cycle. For this purpose, we replaced the endogenous *RAD5* gene with each mutant allele carrying a Myc epitope on the yeast chromosome. These mutant cells synchronously progressing from a G1-block were subjected to Phos-tag western blotting and FACS analyses at each time point. As shown in Fig 4D and 4E, the cell-cycle dependent oscillation of Rad5 protein levels was diminished in *rad5-S130A* and *rad5-S129A S130A* cells compared with wild-type cells. Similar results were also obtained in *rad5Δ* cells expressing Rad5 or Rad5 S129A S130A from plasmids under control of the *RAD5* promoter (S2 Fig). We note that subtle slower-migrating bands detected in *rad5-S130A* were disappeared in *rad5-S129A S130A*, suggesting that only a small portion of Rad5 is constitutively phosphorylated at S129 over cell cycle (Fig 4E). Overall, these results suggest that phosphorylation at S130 but not at S129 plays important role in cell-cycle oscillation of Rad5 and is thus likely to be associated with the cell-cycle-dependent fluctuations in its protein levels.

### Cdc28/CDK1-dependent phosphorylation of Rad5 S130

S130 fits the minimal consensus for phosphorylation by the cyclin-dependent kinase Cdc28/CDK1 (i.e., the presence of a Ser/Thr-Pro moiety). Accordingly, the slow mobility band of Rad5 was largely diminished in samples from *rad5-P131A* cells, which are comparable to *rad5-S130A* cells (Fig 2C). These results imply that Rad5 might be phosphorylated on S130 by CDK1. To investigate Rad5 phosphorylation in cells deficient in CDK1 activity, we employed an ATP analog-sensitive mutant, *cdc28-as1*, which can inactivate CDK1 in the presence of 1NM-PP1 [29]. Asynchronous *cdc28-as1* cells in which Rad5-Myc was expressed from the chromosomal *RAD5* locus were treated for 1 h with dimethyl sulfoxide (mock treatment) or 1NM-PP1; subsequently, samples were analyzed by FACS and Phos-tag western blotting (Fig 4F and S3A Fig). Rad5 phosphorylation was not affected by the mock treatment but was slightly reduced by 1NM-PP1 treatment (Fig 4F, lanes 2 and 3). Since Rad5 phosphorylation levels are likely to be up-regulated during S/G2 phase, asynchronous *cdc28-as1* cells were treated with either HU or nocodazole for 2 h to arrest cell-cycle at S or G2/M phase, followed by further incubation for 1 h with or without 1NM-PP1. In agreement with the posited cell-cycle-dependent Rad5 phosphorylation, we found that Rad5 phosphorylation increased during S- and G2/M-phase arrest and that 1NM-PP1 treatment resulted in a significant decrease in Rad5 phosphorylation levels in S-phase arrested cells but not in G2/M-phase arrested cells (Fig 4F, lanes 6 and 9). A similar behavior was seen in S-phase arrested *rad5-S129A* cells (S3B Fig). These results suggest that Rad5 phosphorylation at S130 is primarily targeted by CDK1 during S phase, and therefore, reaches its maximum in the S/G2 phase. We noted that a low but detectable level of phosphorylation was still observed in the presence of 1NM-PP1 in wild-type and *rad5-S129A* cells. This may be due to the incomplete inactivation of CDK1 activity or another kinase(s) alternatively involved in the S130 phosphorylation.

### Effect of phosphorylation on Rad5 stability

**Fig 5:**
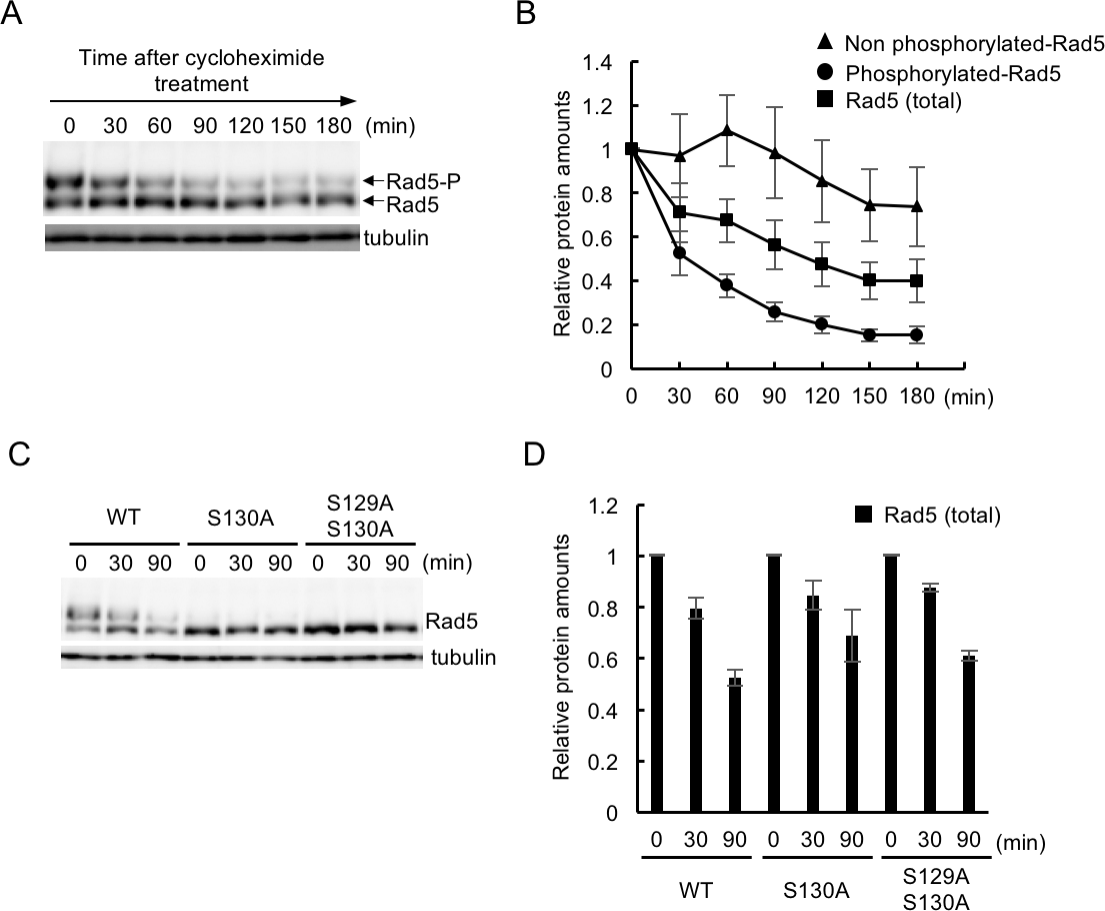
Rapid decay of S130 phosphorylation species. (A) *RAD5-Myc* cells were asynchronously grown in YPDA, and were then treated with CHX (400 μg/ml) for the indicated times to shut off transcription. The stability of Rad5 was examined by Phos-tag western blotting. (B) The band intensities of phosphorylated and unphosphorylated Rad5 shown in (A) were quantified (n = 3). Normalized levels of each band relative to 1.0 at time 0 are shown. Error bars represent the standard errors of the three independent experiments. (C) Cells grown to early log phase at 30°C were transferred into YPDA containing CHX. Samples were obtained at 0, 30, and 90 min after the addition of CHX, and were then subjected to Phos-tag western blotting. (D) The overall band intensity of each lane in (C) was quantified, and the Rad5 protein level remained after the addition of CHX was shown relative to 1.0 at time 0. Error bars represent the standard errors of the three independent experiments.

Since the phosphorylation of S130 affects the cell-cycle-dependent oscillation of total protein levels, we hypothesized that phosphorylation might contribute to Rad5 stability. To test this, we investigated the stability of phosphorylated and non-phosphorylated Rad5 by Phos-tag western blotting of cells treated with cycloheximide (CHX), a protein synthesis inhibitor. We found that the level of total Rad5 protein decreased gradually during CHX treatment with a half-life of ~120 min (Fig 5A and 5B). Interestingly, non-phosphorylated Rad5 species exhibited a relatively long half-life of >180 min, whereas phosphorylated Rad5 rapidly disappeared, with a half-life of approximately 40 min (Fig 5A and 5B). Furthermore, the rapid decrease of phosphorylated Rad5 resulted in a reciprocal increase of non-phosphorylated Rad5 up to 60 min in the presence of CHX, although the latter increase was limited (Fig 5B), suggesting that dephosphorylation of Rad5 is partially responsible for the rapid decrease of phosphorylated Rad5. To further test whether the phosphorylation of Rad5 is required for its rapid turnover, we measured the amount of total Rad5 protein during CHX treatment in wild-type and phospho-deficient *rad5* cells. We found that phosphorylation-deficient Rad5-S130A and Rad5-S129A S130A were more stable than wild-type Rad5 during CHX treatment (Fig 5C and 5D). Thus, dephosphorylation alone would not account for the entire difference in protein stability between wild-type Rad5 and Rad5-S130A or Rad5-S129A S130A. Taken together, these findings demonstrate that the phosphorylation of S130 in part contributes to the degradation of Rad5.

In this study, we identified two target sites for Rad5 phosphorylation: at S130 and S129. S129, a minor phosphorylation site, is constitutively phosphorylated, whereas S130, a major phosphorylation site, is primarily targeted by CDK1 phosphorylation during the S/G2 phase. A genetic analysis of the phosphorylation-deficient mutants suggests that Rad5 phosphorylation is not likely to be involved in DDT, which is consistent with the result that DNA damage does not affect the phosphorylation levels of Rad5. Thus, how cell cycle regulation of Rad5 phosphorylation contributes to the maintenance of genome stability remains unclear at present. It was previously shown that overexpression of Rad5 sensitizes cells lacking Dun1, a DNA damage checkpoint kinase, towards replication-blocking agents like HU [30], implying that uncontrolled protein levels of Rad5 would have adverse effects on the S phase progression. As Rad5 phosphorylation at S130 is cell cycle regulated with a peak at S phase and plays a certain role in the regulation of the total protein levels, it is possible that abrogation of S130 phosphorylation may be potentially detrimental to cell survival and genomic integrity when Rad5 accumulates to deleterious levels under some stressed conditions and/or in a specific cell cycle stage. Further studies should improve our understanding of the connection between Rad5 phosphorylation and DDT activity.

We found that the cell-cycle-dependent oscillations of Rad5 expression levels are attenuated in *rad5-S130A* cells. In addition, the level of phosphorylated Rad5 is reduced faster than that of unphosphorylated Rad5 during CHX treatment, and Rad5-S130A and Rad5-S129A S130A were partially stabilized compared with wild-type Rad5, implying a correlation between the phosphorylation state of Rad5 and its stability. We note that Rad5-S129A S130A and Rad5-S130A are still degraded, and thus are insufficient for fully inhibiting the turnover of Rad5, suggesting that another mechanism may mediate the rapid degradation of Rad5. Thus, extrapolating Rad5 phosphorylation to wild-type cells remains questionable. One possible explanation is that CDK1-dependent Rad5 phosphorylation at S130 facilitates its degradation, and this may account for why phosphorylated Rad5 is preferentially degraded when both phosphorylated and non-phosphorylated forms are present. There are examples linking protein phosphorylation and its stability, such as phosphodegrons, in which phosphorylation facilitates the subsequent ubiquitylation of a substrate [21, 31]. With respect to the structural features of Rad5 S130 and its surrounding residues, the two protein structure prediction programs, PSIPRED (http://bioinf.cs.ucl.ac.uk/psipred/) and IUPred [32], predict that S130 is likely among intrinsically disordered regions (IDRs) (S4 Fig), which are not likely to form a defined three-dimensional structure. It is now well established that IDRs are frequently subjected to post-translational modifications (PTMs), which increase the functional states [33]. Therefore, it may be possible that the phosphorylation of Rad5 at S130 would serve as a signal to promote the interaction and/or recruitment of certain proteases and/or ubiquitination factors, thereby enabling the regulation of Rad5 stability throughout the cell cycle. In this context, we speculate that a mechanism for promoting turnover of phosphorylated Rad5 may be advantageous for protein quality control because the long-lived Rad5 proteins, which are likely to be more phosphorylated than the newly synthesized ones, might be sources of vulnerability. In order to verify this possibility, other PTMs occurring on Rad5 that generate a potential for cross-regulation should be identified.

## Supporting information

S1 Fig Amino acid sequence alignment of the *Saccharomyces cerevisiae* Rad5 N-terminal 114–144 region. Highly conserved residues are indicated by *bold letters*. The asterisk symbols indicate the putative phosphorylation sites.
(TIF)

S2 Fig Cell cycle-dependent expression of Rad5. *rad5Δ* cells were transformed with each of the pRS415 derivatives bearing *RAD5-Myc* or *rad5 S129A S130A-Myc*. Cells grown to early log phase at 30°C in SC-LEU medium were synchronized in G1 with α-factor and released synchronously into the cell cycle. Samples were taken at the indicated time points after release from G1 block. Cells were fixed in 70% ethanol, and DNA contents were determined by FACS analysis (upper panel). Rad5 protein was analyzed by Phos-tag western blotting (lower panel). Tubulin served as a loading control.
(TIFF)

S3 Fig Cdc28-dependent phosphorylation of Rad5. (A) *cdc28-as1 RAD5-Myc* cells were grown to log phase or arrested at S phase with 200 mM HU or at G2 phase with 20 μg/ml nocodazole. After 2 h, cultures (pre-treatment) were divided equally and treated with DMSO (mock) or 5 μM 1NM-PP1 for 1 h. Cells were fixed in 70% ethanol and subjected to FACS analysis. Asn denotes asynchronously growing cells. (B) *cdc28-as1 rad5Δ* cells were transformed with each of the pRS415 derivatives bearing *RAD5-Myc* or *rad5 S129A-Myc*. Cells were grown to log phase and then arrested in S phase by addition of HU (200 mM). After 2 h, cultures (pre-treatment) were divided equally and treated with DMSO (mock) or 5 μM 1NM-PP1 for 1 h. DNA content was determined by FACS (upper panel). Rad5 protein was analyzed by Phos-tag western blotting (lower panel). Tubulin served as a loading control.
(TIFF)

S4 Fig Intrinsic disorder profile of Rad5 N-terminal region. (A) Disorder tendency of Rad5 N-terminal sequence (1–200 amino acid residues) are shown as confidence score, using the PSIPRED protein sequence program (DISOPRED). The position of the corresponding serine 130 residue is indicated by arrow. (B) A schematic representation of the secondary structure map of Rad5 N-terminal region. Feature predictions by DISOPRED are color coded onto the sequence according to the sequence feature key shown below.
(TIFF)
