# Supplementary figures and images for: Cyclin-dependent kinase modulates budding yeast Rad5 stability during cell cycle
Source: PLoS One. 2018 Sep 26;13(9):e0204680. doi: 10.1371/journal.pone.0204680 (PMC6157869; doi:10.1371/journal.pone.0204680)

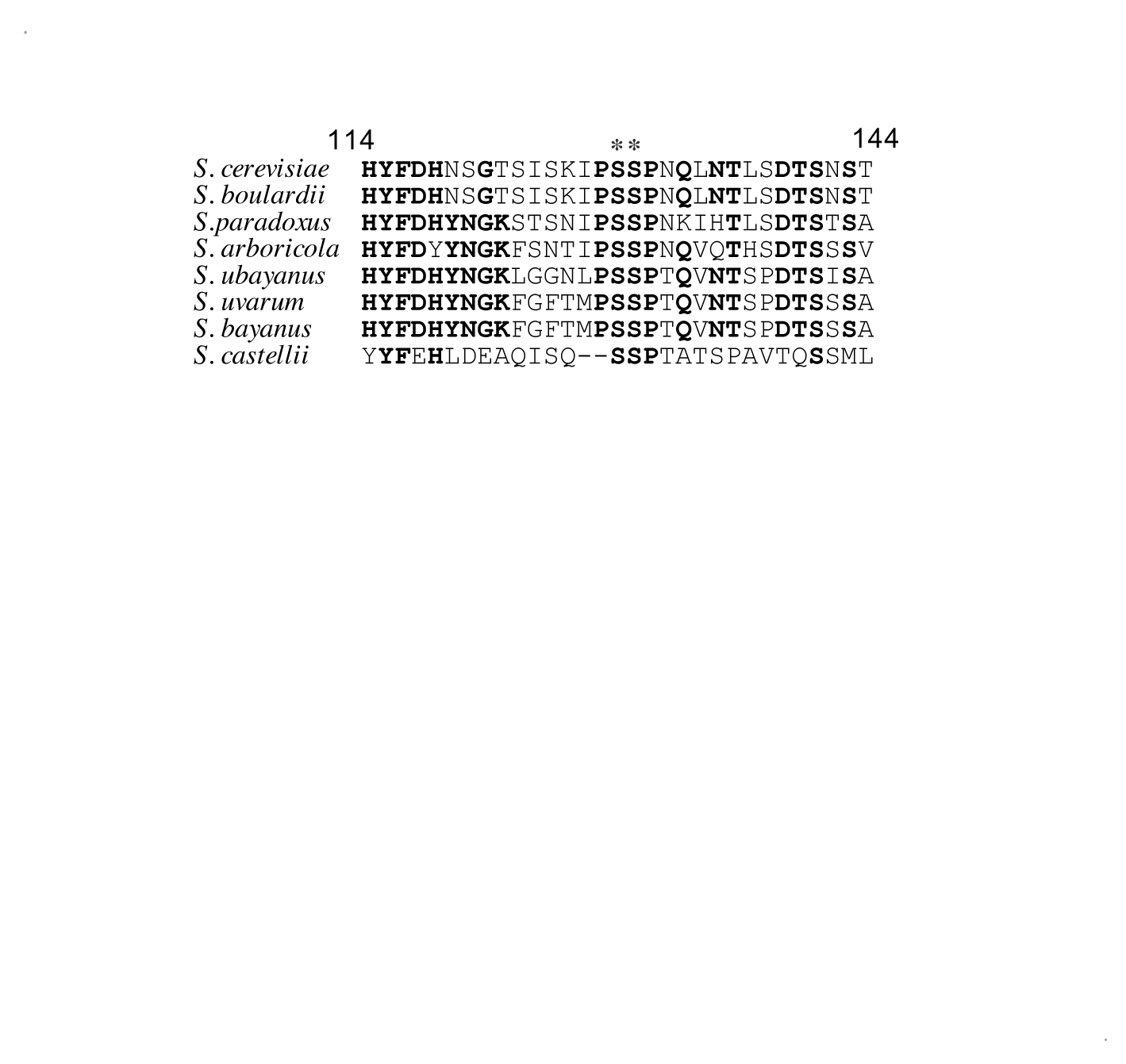

Supplement: S1 Fig — Highly conserved residues are indicated by bold letters. The asterisk symbols indicate the putative phosphorylation sites. (TIF) [file pone.0204680.s001.tif]

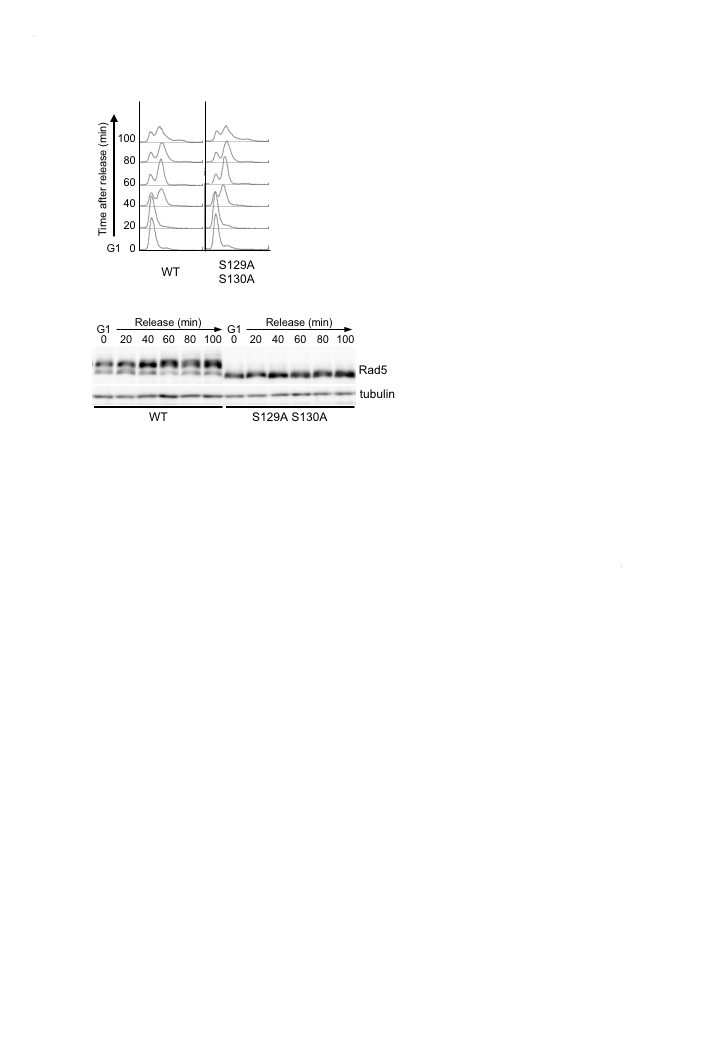

Supplement: S2 Fig — rad5Δ cells were transformed with each of the pRS415 derivatives bearing RAD5-Myc or rad5 S129A S130A-Myc. Cells grown to early log phase at 30°C in SC-LEU medium were synchronized in G1 with α-factor and released synchronously into the cell cycle. Samples were taken at the indicated time points after release from G1 block. Cells were fixed in 70% ethanol, and DNA contents were determined by FACS analysis (upper panel). Rad5 protein was analyzed by Phos-tag western blotting (lower panel). Tubulin served as a loading control. (TIFF) [file pone.0204680.s002.tiff]

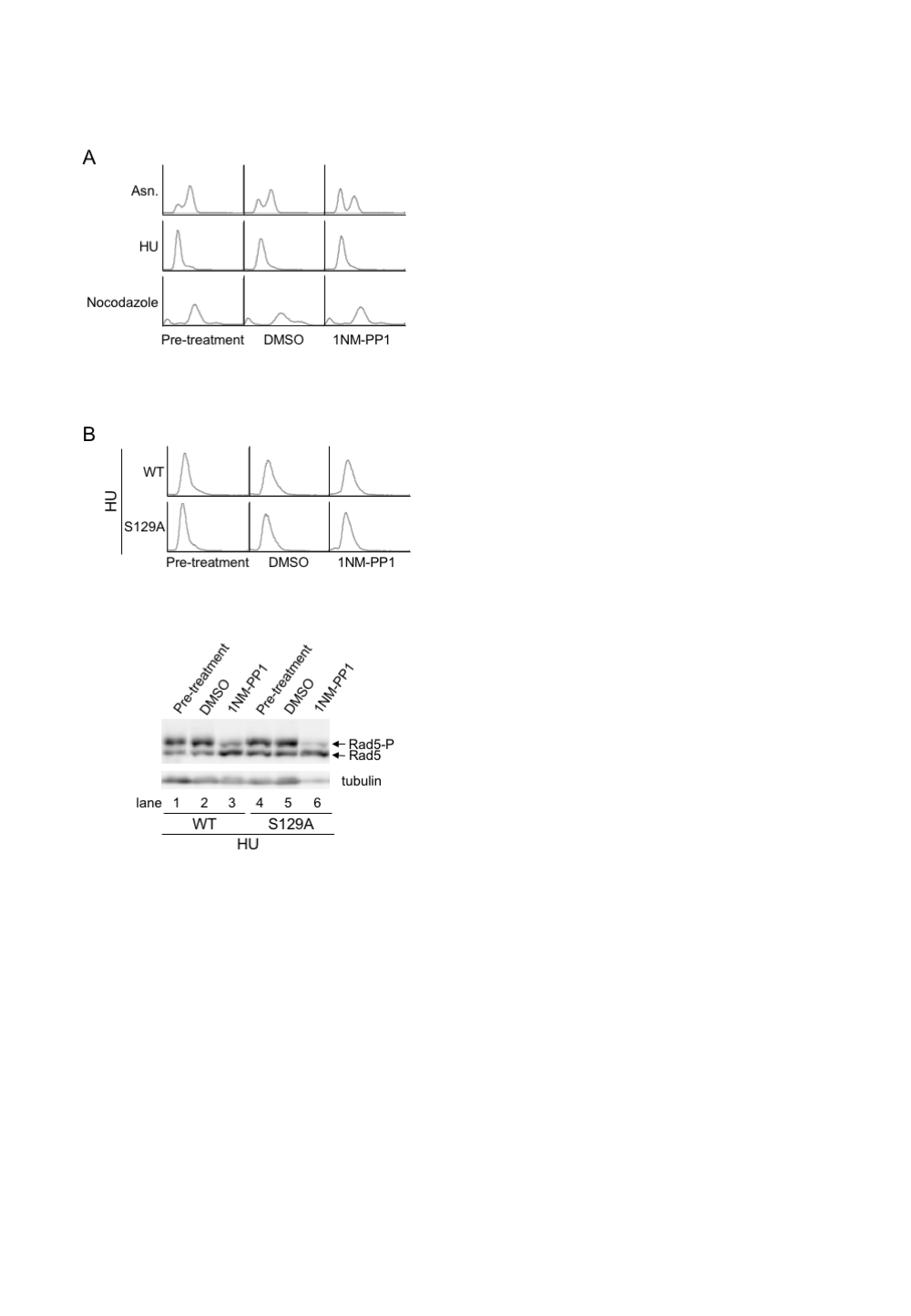

Supplement: S3 Fig — (A) cdc28-as1 RAD5-Myc cells were grown to log phase or arrested at S phase with 200 mM HU or at G2 phase with 20 μg/ml nocodazole. After 2 h, cultures (pre-treatment) were divided equally and treated with DMSO (mock) or 5 μM 1NM-PP1 for 1 h. Cells were fixed in 70% ethanol and subjected to FACS analysis. Asn denotes asynchronously growing cells. (B) cdc28-as1 rad5Δ cells were transformed with each of the pRS415 derivatives bearing RAD5-Myc or rad5 S129A-Myc. Cells were grown to log phase and then arrested in S phase by addition of HU (200 mM). After 2 h, cultures (pre-treatment) were divided equally and treated with DMSO (mock) or 5 μM 1NM-PP1 for 1 h. DNA content was determined by FACS (upper panel). Rad5 protein was analyzed by Phos-tag western blotting (lower panel). Tubulin served as a loading control. (TIFF) [file pone.0204680.s003.tiff]

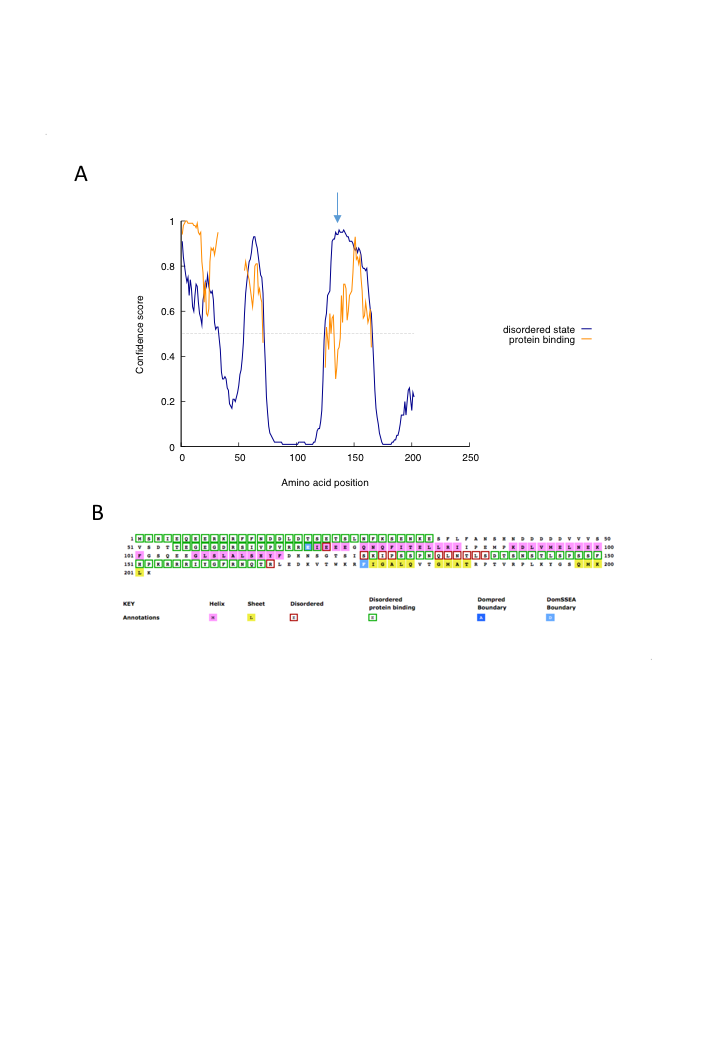

Supplement: S4 Fig — (A) Disorder tendency of Rad5 N-terminal sequence (1–200 amino acid residues) are shown as confidence score, using the PSIPRED protein sequence program (DISOPRED). The position of the corresponding serine 130 residue is indicated by arrow. (B) A schematic representation of the secondary structure map of Rad5 N-terminal region. Feature predictions by DISOPRED are color coded onto the sequence according to the sequence feature key shown below. (TIFF) [file pone.0204680.s004.tiff]
